# Expansion of bovine skeletal muscle stem cells from spinner flasks to benchtop stirred-tank bioreactors for up to 38 days

**DOI:** 10.3389/fnut.2023.1192365

**Published:** 2023-08-07

**Authors:** Dimitrios Tzimorotas, Nina Therese Solberg, R. Christel Andreassen, Panagiota Moutsatsou, Vincent Bodiou, Mona Elisabeth Pedersen, Sissel Beate Rønning

**Affiliations:** ^1^Nofima AS, Food Safety and Health, Ås, Norway; ^2^Nofima AS, Raw Materials and Process Optimization, Ås, Norway; ^3^Mosa Meat BV, Maastricht, Netherlands

**Keywords:** skeletal muscle satellite cell, stem cell expansion, bioreactor, microcarriers, upscaling

## Abstract

**Introduction:**

Successful long-term expansion of skeletal muscle satellite cells (MuSCs) on a large scale is fundamental for cultivating animal cells for protein production. Prerequisites for efficient cell expansion include maintaining essential native cell activities such as cell adhesion, migration, proliferation, and differentiation while ensuring consistent reproducibility.

**Method:**

This study investigated the growth of bovine MuSC culture using low-volume spinner flasks and a benchtop stirred-tank bioreactor (STR).

**Results and discussion:**

Our results showed for the first time the expansion of primary MuSCs for 38 days in a bench-top STR run with low initial seeding density and FBS reduction, supported by increased expression of the satellite cell marker PAX7 and reduced expression of differentiation-inducing genes like MYOG, even without adding p38-MAPK inhibitors. Moreover, the cells retained their ability to proliferate, migrate, and differentiate after enzymatic dissociation from the microcarriers. We also showed reproducible results in a separate biological benchtop STR run.

## 1. Introduction

The livestock industry worldwide is under increasing pressure to meet the growing demand for high-quality animal protein. A revolutionary new alternative to conventional animal protein production is cultivating muscle stem cells outside the live animal in a bioreactor, bypassing animal husbandry. Optimistic estimates suggest 10,000 kg of edible animal protein can be obtained from just 1 g of bovine muscle using this modern technology (1). The process involves the following steps: (1) biopsy collection and isolation of skeletal muscle satellite cells (MuSCs), (2) multiplication of cells in a bioreactor (expansion), (3) differentiation of MuSCs into muscle cells and myofibers, and (4) assembly into a final food product (2). MuSCs used in cultured animal protein production are genetically variable primary cells, usually isolated directly from animal tissues (3), and retain their morphological and functional characteristics from the tissue of origin (4). A disadvantage of using MuSCs is that they enter the cellular senescence phase and are limited to < 50 doublings (5, 6). Cellular senescence is a substantial limitation in stem cell therapies during tissue reconstruction as it results in the inability of undifferentiated MuSCs to expand in culture while maintaining transplantation potential (7). Similarly, large-scale production of edible animal proteins depends on a high self-renewal and proliferation capacity.

The methodology for culturing animal proteins is inspired by techniques used for medical purposes such as the reconstruction of damaged muscle tissue and the production of biopharmaceuticals (8, 9). MuSCs are adherent cells, and efficient expansion of cells requires a high volume-to-surface area. This can be achieved using a static cell culturing approach with a multi-layer vessel that allows cells to grow in stacked layers, an approach that is used to produce the first cultured burger in 2013 (10). However, the available surface area in monolayer systems is restricted, which limits scalability and introduces space constraints due to the low surface area-to-volume ratio. Growing cells in dynamic bioreactor systems have emerged as a viable approach to further increase the efficiency for upscaled expansion. This bioprocess is widely used to expand human mesenchymal stem cells (hMSCs) in fully controlled bioreactors (11, 12). Most studies on the expansion of hMSCs have been performed in stirred-tank bioreactors (STRs) using microcarriers, and now, suitable commercially available STR systems exist ranging from 0.5 to 6000 L (13, 14). Lawson et al. demonstrated that human mesenchymal stromal cells could be expanded 43-fold in 50 L bioreactors (15) (16). Although bioreactors for large-scale production of biopharmaceuticals using mammalian cells are widely available and many commercial systems have been developed to facilitate and intensify the process, this technology still needs to be optimized before it can be used for the production of edible animal proteins (14). Tissue culture techniques are still small-scale (tissues produced are in mm to cm range at most) and are rather costly. The key to successful upscaling of cell expansion processes depends on many factors, and each bioreactor culture system has advantages and disadvantages (14, 17). Packed bed bioreactors and hollow fiber bioreactors protect the cells from hydrodynamic forces, allow good oxygenation through fluidized media and efficient recycling of cell growth media, and have been used in the expansion of the mouse C2C12 skeletal muscle cell line (14). These methods might, however, face upscaling challenges due to the formation of pH, dO, and nutrient gradients because of the lack of mixing. MuSC cultivation through aggregation into spheroids or adherence to microcarrier STRs can be used to improve the scalability. Spheroid formation facilitates cell–cell communication, delays senescence, and allows the cells to establish a more 3D-like network (13). There might be challenges which include a lack of control of aggregate sizes and cell densities, and the functional size of spheroids is limited to 300 μM (13). Microcarrier-based STRs benefit from a much larger surface area-to-volume ratio compared with monolayered cultures (17). When MuSCs are grown in suspension on microcarriers (MCs), a gas flow and agitation system, such as a stirrer, ensures adequate mixing of nutrients and gasses. The performance of the STR is dependent on many factors, such as the size and shape of the stirrer, the aeration system, the ratio of liquid height to tank diameter, and the shape of the vessel (13). Once the MuSCs have reached a high density, new MCs are added to the STR to provide more surface area for the cells to colonize and continue proliferating (18–20). STRs have the advantage of a more homogeneous environment over static culture vessels, but adequate oxygenation must be balanced against the adverse effects of hydrodynamic forces on the cells (14). In addition, nutrient utilization and waste production must be monitored to ensure efficient cell expansion and control its effects on the cell phenotypic stage (quiescence, proliferation, and differentiation). Sustaining proliferation of MuSCs is an important prerequisite for cell expansion and requires an optimal growth medium with carbon sources (usually glucose), salt, amino acids, and factors that promote cell attachment, migration, and growth. MuSCs can be successfully expanded on microcarriers in spinner flask cultures (21, 22). Currently, no studies have been published on the expansion of primary bovine MuSCs in a controlled STR for long-term expansion, although Boudreault et al. have demonstrated the expansion of human MuSCs in spinner flasks up to 500 ml for 20 days where cells retained therapeutic efficiency when transplanted into mice (23), and Hanga et al. have also lately demonstrated a successful 11-day cultivation of bovine adipose-derived stem cells in a 3L benchtop STR, for the production of cultivated fat (24).

Previous studies on bovine MuSCs have been performed in spinner flasks, with limited control of temperature, pH, oxygenation, and nutrient consumption (21). Furthermore, these studies were conducted over a relatively short period of time (8–9 days), with limited cell multiplication. This study aims to investigate the ability of MuSCs to expand for a longer period (38 days) in an STR using low cell seeding density, low glucose, and low serum content in a cell culture medium, without compromising the MuSC quality.

## 2. Materials and methods

### 2.1. Cell culture materials

Gibco™ Dulbecco's Modified Eagle Medium (DMEM) low glucose supplemented with GlutaMAX™ and pyruvate, fetal bovine serum (FBS), and penicillin/streptomycin solution 10,000 units ml−1 (P/S), amphotericin B 250 μg ml−1, PBS, and 0.05% trypsin/EDTA was purchased from Thermo Fisher Scientific (Waltham, MA, USA). Entactin-Collagen-Laminin (ECL) was purchased from Millipore Sigma (Burlington, MA, USA), and bovine skin collagen was purchased from Sigma–Aldrich (BioReagent). Dimethyl sulfoxide (DMSO), collagenase, and Cytodex^®^1 microcarrier were purchased from Sigma–Aldrich (Merck KGaA, Darmstadt, Germany). Ultroser™ G was purchased from PALL Corporation (NY, USA).

### 2.2. Isolation of bovine MuSCs

Bovine MuSCs were isolated from newly slaughtered cattle at an industrial abattoir using a well-established method (25, 26). The cells were extracted from Longissimus thoracis (beef sirloin, Nortura AS, Rudshøgda, Norway), using nine animals of the same age (young animals), gender (bulls), and breed (Norwegian Red). Animal procedure approval was not required according to the Norwegian Food Safety Authority and the Norwegian Law. In short, one muscle biopsy sample (1–2 g) from each animal was digested with 0.72 mg ml−1 collagenase in 10 ml DMEM containing 500 units P/S and 12.5 μg amphotericin B and shaken for 1 h at 37 °C at 70 rpm. The tissue was further digested with 0.05% trypsin/EDTA for 25 min, followed by the addition of 10% FBS for enzyme inactivation. This step was repeated three times before the cells were pooled. To purify the MuSCs population (i.e., fibroblast removal), the cell suspensions were incubated in uncoated 25 cm^2^ cell culture flasks for 1 h at 37°C and 5% CO_2_ in 3 ml cell culture medium (DMEM) supplemented with 10% FBS, 3.75 μg amphotericin B, and 150 units P/S. Fibroblasts adhered to the plastic, and the non-adhering MuSCs were then collected and seeded onto 25 cm^2^ cell flasks coated with 1 mg ml−1 Entactin-Collagen-Laminin (ECL) until 50% confluency. The isolated MuSCs were then collected, transferred into 75 cm^2^ ECL-coated culture flasks until 70–80% confluence, and stored in freezing medium (1.5 ml DMEM containing 8% DMSO and 1 ml FBS) in liquid nitrogen until further use. This isolation procedure results in a cell population of more than 90% MuSCs with the ability to proliferate and differentiate (25). After thawing, the cells were cultivated at 37°C and 5% CO_2_ in cell growth culturing medium containing DMEM medium supplemented with 2% FBS, 2% Ultroser G, and 1.25 μg ml−1 amphotericin B, and 50 units per ml P/S. Ultroser G is a serum substitute that allows the reduction of the amount of FBS needed for cell growth and as such reduces the effects of the lot-to-lot serum variability often observed while using FBS. Ultroser G serum substitute has a potency five times higher than FBS (i.e., 2 % of reconstituted Ultroser G serum substitute is equivalent to 10% FBS in the basal medium). The experiments were preferably performed with cells at passage 6.

### 2.3. Preparation of cytodex^®^1 microcarriers

Cytodex^®^1 or Cytodex^®^3 microcarriers were prepared according to the manufacturer's instruction. In short, microcarriers (MCs) were transferred to falcon tubes and left to swell in PBS for 3.5 h, before being washed twice with PBS. The MCs were then autoclaved at 121°C for 15 min before PBS was removed, and the MCs were rinsed in a cell culture medium.

### 2.4. Expansion of MuSCs in spinner flasks and benchtop stirred-tank bioreactor

**Figure 1:**
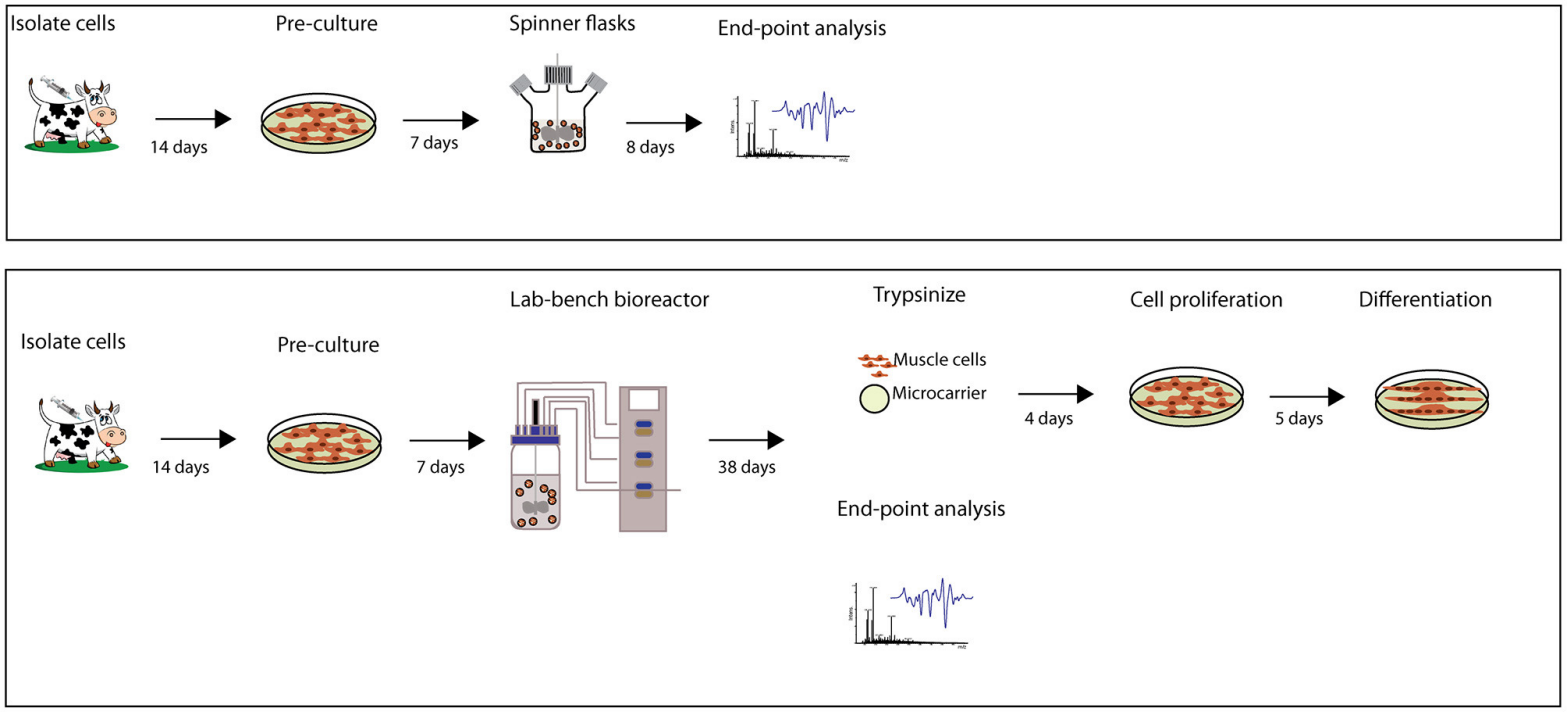
Flowchart describing the workflow of the experiments with approximate timelines. Bovine MuSCs were isolated from newly slaughtered cattle at an industrial abattoir using a well-established method. Cells were pre-cultured in the planar system for approximately 7 days until we reached high enough cell densities to initiate the spinner flask or lab-bench bioreactor experiments.

We conducted two different approaches for the expansion of MuSCs. Figure 1 presents the experimental design using either spinner flasks for 8 days or benchtop stirred-tank bioreactor for 38 days. Spinner flasks were used to determine whether MuSCs could be expanded in spinner flasks using low cell seeding density at the initial starting point in a cultivation medium containing 2% FBS and 2% Ultroser G, with 9,000 cells/ml (1,800 cells/cm^2^ MC) at 38°C. Spinner flasks with high serum (10% FBS + 2% Ultroser G, serum concentrations equivalent to GM often used for bovine MuSCs (27, 28)) cultivation medium and high cell density (20,000 cells/ml and 900 cells/cm^2^ MC at 37°C) were used for comparison. Spinner flask cultures were run in biological triplicates with cells isolated from two different donor animals, using 100 ml cultivation media without antibiotics at 50 rpm stirred for 8 days. DWK Life Sciences Wheaton™ Magna-Flex™ spinner flasks (250 ml, Fisher Scientific, MA, USA) were used. The day 0 sampling was performed after 20 min of inoculation (no stirring).

Two independent biological replicate experiments were performed in the benchtop stirred-tank bioreactor (BioFlo^®^ 120 benchtop stirred-tank bioreactor, Eppendorf, AG, Hamburg, Germany), using MuSCs isolated from different bovine donors. The cells were seeded out in one replicate for each run, using cells isolated from different donor animals. For run 1, cells were isolated and pooled from five donor animals, while for run 2, cells were isolated from one donor animal. The following seeding conditions were used: 1,800 cells were seeded per cm^2^ MC, i.e., 6.3 million MuSCs combined with 0.8 g of MCs in 700 ml cultivation medium without antibiotics were added to the BioFlo^®^ 120 benchtop stirred-tank bioreactor. The cell cultivation medium contained DMEM, 2% FBS, and 2% Ultroser G. The pH was set to 7.3 and maintained using a selected combination of overhead gases (5 % CO_2_, 95% compressed air). The temperature was set to 38°C and was controlled using a water bath/chiller (DASGIP^®^ Recirculating Chiller, Eppendorf, Hamburg, Germany). Cell attachment on the MCs was facilitated by no mixing for 1 h, followed by continuous agitation at 40 rpm using a pitched blade impeller (Eppendorf, AG, Hamburg, Germany). On each sampling day, 7 ml of sample was removed from the middle of the vessel and split for subsequent analysis as described below. Day 0 sampling was performed 20 min after inoculation. To keep the cell culture medium at a low cost, to supply new nutrients, and to eliminate waste products produced by the cells (based on our media analysis for metabolites), we performed an exchange volume of 30%. The experiment was conducted with two separate runs, and the following actions were performed during the runs: run 1: 30% cultivation medium was exchanged after sampling and sedimentation of microcarriers and cells on day 8 (200 ml cultivation medium removed and 300 ml fresh added), day 13 (250 ml cultivation medium removed and 300 ml fresh added), and day 27 (150 ml cultivation medium removed and 200 ml fresh added). In total, 0.4 g Cytodex^®^1 microcarriers were added on days 13 and 27; run 2: 30% of cultivation medium was exchanged after sampling and sedimentation of microcarriers and cells on days 8, 14, and 28. Overall, 0.4 g Cytodex^®^1 microcarriers were added on days 14 and 28. The agitation was temporarily paused during the medium exchange.

### 2.5. Cell growth quantification

The DNA concentration of cultivated MuSCs in run 1 was measured using Quant-IT Picogreen ds DNA Assay (Fluorescence, Invitrogen, Paisley, UK). In brief, 1 ml of MC/cell suspension sample was washed with PBS and then dissolved in an RLT lysis buffer (Qiagen GmbH, Venlo, The Netherlands). The solution was then incubated at 55^o^C for 15 min and vortexed. The MCs were sedimented, and 1 μl of the supernatant was added to 99 μl 1xTE into a 96-well plate and mixed four times using a multichannel pipette. A standard curve was prepared by adding 6 μl DNA standard (100 ng/μl) to 594 μl 1xTE and serial dilutions. In total, 100 μl of each standard in duplicates was added to a 96-well black fluorometer plate. Overall, 100 μl 1:200 dilution of Picogreen was added to each well and mixed four times with a multi pipette followed by 5 min of incubation in the dark. The fluorescence signal was measured using a Synergy H1 hybrid multimode reader (BioTek Instruments Inc., Winooski, VT, USA). The average DNA concentration of parallels was calculated using the standard curve. To express the cell growth as cells/ml, we correlated DNA concentration with day 0 measurements. On day 0, the DNA measurement was 2.5 ng/μl, which theoretically corresponds to 9 × 10^3^ cells/ml (initial cell seeding density). For each time point, the cell density in cells/ml was calculated based on measured DNA, and a growth curve was generated.

### 2.6. Glucose measurements

Glucose concentration in the MuSCs culture medium was measured using the Reflectoquant test strips prepared by Merck KGaA (Darmstadt, Germany). The method is based on the conversion of glucose into gluconic acid lactone, under the catalytic effect of glucose oxidase. The hydrogen peroxide formed, in the presence of peroxidase, reacts with an organic redox indicator to form a blue-green dye that is determined reflectometrically using the RQflex 10 instrument from Merck KGaA (Darmstadt, Germany). Reflectometric test strips were first dipped into a suitably diluted supernatant of the samples (measuring concentration range is between 1 and 100 mg/L) and then carefully placed on the RQflex10.

### 2.7. Lactate measurements

Lactate production from the MuSCs into the cultivation medium was measured using the Reflectoquant test strips prepared by Merck KGaA (Darmstadt, Germany). The method is based on the oxidation of lactate by nicotinamide adenine dinucleotide (NAD) under the catalytic effect of lactate dehydrogenase to pyruvate. In the presence of diaphorase, NADH formed in the process reduces tetrazolium salt to a blue formazan that is determined reflectometrically using the RQflex 10 instrument. The reflectometric test strips were first dipped into a suitably diluted supernatant of the samples (measuring concentration range is between 3 and 60 mg/L) and then carefully placed on the RQflex10.

### 2.8. Immunocytochemistry and light microscopy

Hoechst stain (1 drop NucBlue, live cell stain probe Hoechst33342) was added to 500 μl MCs/cell suspension sample, followed by 20 min of incubation in the dark at RT. The MCs were sedimented in an Eppendorf tube, and the supernatant was then removed before the MC/cells were washed with 1 ml of PBS. After re-settling, the MC/cells were suspended in 100 μl PBS and transferred onto a microscope slide. The MC/cells were examined using a fluorescence microscope (ZEISS Axio Observer Z1 microscope), and the images were processed using Adobe Photoshop CS3. For immunofluorescence staining of bovine MuSCs on MCs, the MC/cell suspension was washed with PBS and fixed with 4% PFA for 10 min at RT. The MC/cell suspension was then washed twice with PBS, permeabilized with 0.1% Triton-X-100 for 5 min, washed 1x with PBS, and left in 1x blocking buffer (ab126587) in PBS-tween for 30 min, followed by incubation with the primary antibody for 1 h and diluted in 0.1x blocking buffer in PBS-tween. Subsequent incubation was performed for 30 min with the secondary antibodies, together with Alexa Fluor™ 488 Phalloidin (1:200) and NucBlue Live Cell-stained Ready Probe (Invitrogen). Then the MC/cell suspension was transferred onto a microscope slide and mounted using the DAKO fluorescent mounting medium (Glostrup, Denmark). The MC/cells were examined using the fluorescence microscope (ZEISS Axio Observer Z1 microscope), and the images were processed using Adobe Photoshop CS3. Mouse anti-NCAM/CD56 5.1H11, 1:10 was purchased from the Developmental Studies Hybridoma Bank (Iowa City, IA, USA). Mouse anti-α-Tubulin T5168 1:400 was purchased from Sigma–Aldrich. Alexa 546-conjugated goat anti-mouse was purchased from Thermo Fisher Scientific (Waltham, MA, USA).

### 2.9. RNA extraction and real-time qPCR

To analyze the gene expression of bovine MuSCs during expansion, 1–2.5 ml of MC/cell suspension was centrifuged at 300 *g* for 5 min, followed by careful removal of the supernatant, which was washed once with PBS. Total RNA was obtained using an RNeasy Mini Kit [50] (Qiagen, Hilden, Germany), according to the manufacturer's instructions, including 20 μl 2M DTT per ml RLT buffer and a DNase step. cDNA was generated using a SuperScripct VILO cDNA synthesis kit (Invitrogen, Carlsbad, CA, USA) according to the manufacturer's protocol. RT-qPCR was carried out using TaqMan Gene Expression Master Mix and QuantStudio5 (Applied Biosystems, Foster City, CA, USA) PCR System. The amplification protocol was initiated for 2 min at 50°C, followed by denaturation for 10 min at 95°C, then 45 cycles of denaturation for 15 s at 95°C, annealing of TaqMan probes, and amplification at 60°C for 1 min. RT-qPCR analyses were performed on two biological replicates, each with three technical replicates. Gene expressions were normalized to the *EEF1A1* internal control, and the bars indicate fold change relative to the day 0 control for each gene analyzed. Table 1 show the primers/probes analysed with RT-PCR.

**Table 1:** Gene target and TaqMan^®^ primer/probe assays.

**Gene target**	**TaqMan^^®^^ primer/probe assays**
*EEF1A1*	Bt03223794_gl
*PAX7*	Hs00242962_ml
*MYOD1*	Bt03244740_ml
*MYOG*	Bt03258928_ml
*CCND1*	Bt03235028_ml
*MYF5*	Bt03223134_m1
*DESMIN*	Bt03254510_m1

### 2.10. Determination of metabolic activity (ATP) and cytotoxicity (LDH release)

The metabolic activity (amount of ATP produced) of bovine MuSCs for run 1 was determined using a CellTiter-Glo^®^ Luminescent Cell Viability Assay (Promega, Madison, WI, USA). Cytotoxicity of run 1 was measured as lactate dehydrogenase (LDH) released into the cell culture medium and was performed according to the manufacturer's protocol (Roche Applied Science, Mannheim, Germany). Luminescence and absorbance signals were measured using a Synergy H1 Hybrid multimode microplate reader (Biotek, Winooski, VT, USA). The experiments were performed in technical triplicate, and data are presented as mean ± SEM.

### 2.11. Sub-cultivation of bovine MuSCs in 2d following long-term expansion in a benchtop stirred-tank bioreactor

To measure the proliferative capacity of the bovine MuSCs after long-term expansion in the stirred-tank bioreactor, 12 ml of MC/cell suspension sample was washed once with PBS, before dissociation in 10 ml trypsin for 30 min at 37°C. The cells were washed once in PBS before centrifugation and subsequently resuspended in growth medium and seeded on ECL-coated 6-well plates. The cells were cultured for 4 days, and images were captured every day to monitor cell proliferation. The images were captured using Leica DMIL Led Light Microscope. To determine the migration of cells from MCs, 1 ml of MC/cell suspension was left in ECL-coated chamber slides for 24 h before staining with Alexa Fluor™ 488 Phalloidin (1:200) and NucBlue Live Cell-stained Ready Probe (Invitrogen) for 30 min. To determine myotube formation, proliferating MuSCs were washed with PBS and placed in a differentiation medium (DMEM, 2 % FBS, 1.25 μg ml−1 amphotericin B, 50 units per ml P/S, and 25 pmol Insulin) for 5 days to induce myogenesis.

### 2.12. Data treatment and statistical analysis

Significant variance by treatments was determined either by (1) two-way ANOVA, using Šídák hypothesis testing, or (2) one-way ANOVA using Dunnett's multiple comparison test. Differences were considered significant at a *p*-value of < 0.05. All statistical analyses were performed in GraphPad Prism version 10.0.0 (GraphPad Software, La Jolla, CA, USA).

## 3. Results

### 3.1. Expansion of MuSCs in spinner flasks for 8 days

**Figure 2:**
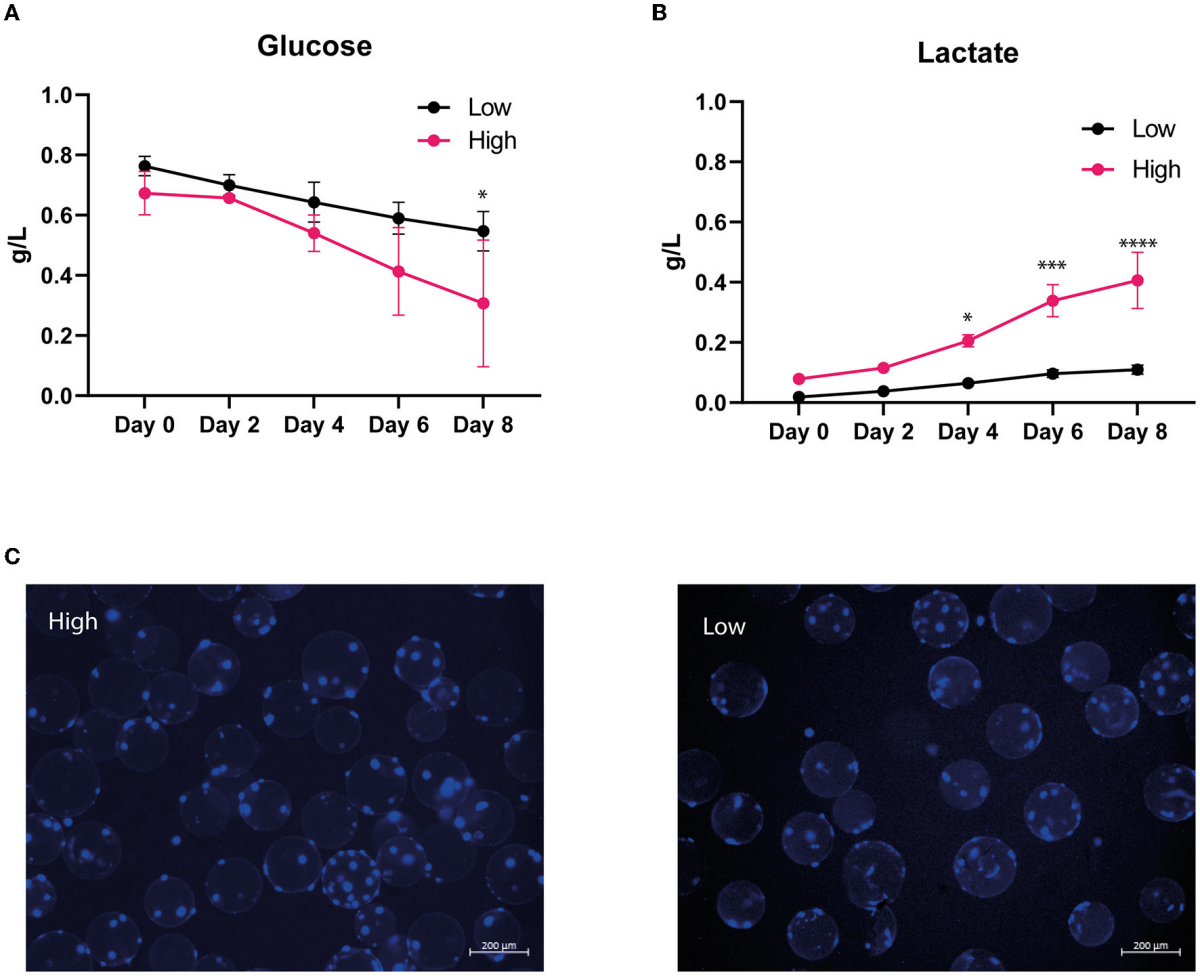
MuSC expansion in spinner flasks. Bovine MuSCs were seeded in biological triplicates on Cytodex^®^1 microcarriers and expanded for 8 days, isolated from two different donor animals. The cells were either seeded in high serum culture medium (10% FBS and 2% Ultroser G) with initial seeding density of 20,000 cells/ml using 900 cells/cm^2^ MC at 37°C or low serum culture medium (2% FBS and 2% Ultroser G) containing 9,000 cells/ml using 1,800 cells/ cm^2^ MC at 38°C. Graphs show the concentrations of glucose **(A)** and lactate **(B)** for the high and low seeding conditions in the spinner flask. Asterisks denote significant differences between high and low seeding conditions at indicated timepoints (^*^ < 0.05, ^***^ < 0.001, and ^****^ < 0.0001, statistics assessed using the two-way ANOVA using Šídák hypothesis testing). **(C)** Evaluation of the cell distribution per MC on day 8. Live cells were stained using NucBlue Live stain, and images were captured using a ZEISS Axio Observer Z1 microscope.

The choice of MCs could affect cell attachment, and our preliminary results showed a higher number of cells/MCs and increased cell growth after 6 days when cells were seeded on Cytodex©1 MCs compared with Cytodex3 (Supplementary Figure S1). Cytodex©1 MCs were, therefore, used for successive experiments. The serum replacement Ultroser G, routinely used in our laboratory for bovine MuSCs cultivation, was also used (25, 26). Spinner flasks were used to determine whether MuSCs could be expanded when seeded at a low initial cell seeding concentration (9,000 cells/ml) and in a cultivation medium with only 2% FBS and 2% Ultroser G (Figure 2). Throughout the bioreactor culture, we examined the glucose concentration in the cell culture media, which indicates the efficiency of cell growth (Figure 2A). In addition, the lactate concentration was measured, aiming to detect the amount of waste metabolites produced from the cells (Figure 2B). During the experiment, glucose was consumed and lactate was produced, and the glucose consumption and lactate production were more prominent with high serum in the cultivation medium/high initial cell seeding. Furthermore, through visual inspection, most MCs contained more than three cells after 8 days in spinner flask culture, even with low serum/low cell seeding concentrations (Figure 2C). This demonstrates that bovine MuSCs tolerate cultivation in spinner flasks using cell culture medium with low serum concentration and with serum replacement. The low serum/low initial seeding density condition was chosen for the following experiments in a lab-bench bioreactor.

### 3.2. Expansion of MuSCs in a lab-bench bioreactor for 38 days

**Figure 3:**
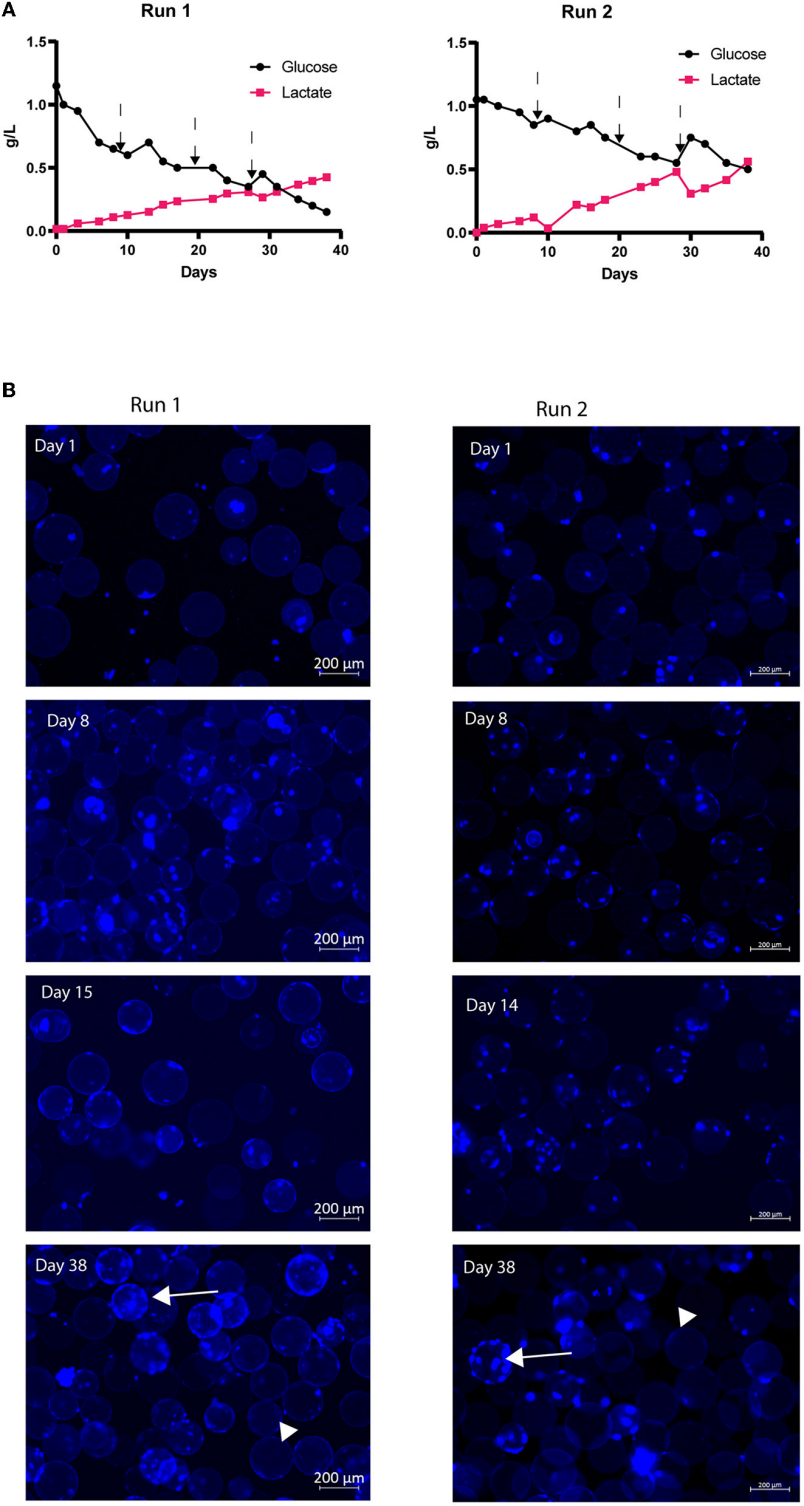
Long-term expansion of bovine MuSCs (38 days) in the benchtop stirred-tank bioreactor, in two biological separate replicate runs. The cells were seeded out in one replicate for each run, using cells isolated from different donor animals. For run 1, cells were isolated from five animals and pooled, while for run 2, cells were isolated from one animal. Skeletal MuSCs were mixed with Cytodex^®^1 microcarrier at a concentration of 9,000 cells/ml (1,800 cells/cm^2^ MCs) in 700 ml of cell growth medium. Run 1: Cell growth medium was changed on days 8, 17, and 27 after sampling, while new microcarriers were added on days 13 and 27, after sampling. Run 2: Cell growth medium was changed on days 8, 14, and 28 after sampling, while new microcarriers were added on days 14 and 28, after sampling. The addition of fresh medium is indicated with arrows. **(A)** Glucose and lactate concentrations during the experiment for run 1 (left) and run 2 (right). **(B)** Cell distribution on MCs during the expansion. Live cells were stained using NucBlue Live stain, and images were captured using a ZEISS Axio Observer Z1 microscope. Arrows indicate MCs with cells, arrowheads: empty MCs.

**Figure 4:**
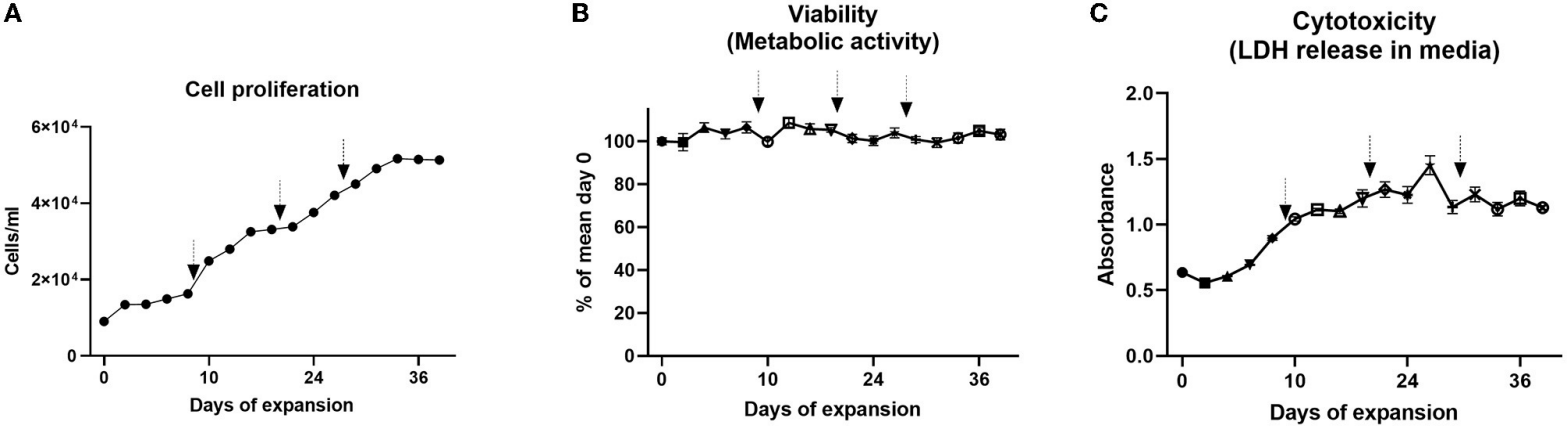
Long-term expansion of bovine MuSCs (38 days) in the lab-bench stirred tank bioreactor (run 1). The cells were seeded out in one replicate for each run using pooled cells isolated from five different donor animals. **(A)** Cells/ml based on DNA concentration values. To express the cell growth as cells/ml, we correlated DNA concentration with day 0 measurements. On day 0, the DNA measurement was 2.5 ng/μl, which theoretically corresponds to 9x10^3^ cells/ml (initial cell seeding density). For each time point, the cell density in cells/ml was calculated based on measured DNA, and a growth curve was generated. **(B)** Cell metabolic activity (ATP production) was normalized to the mean of technical triplicate values on day 0. **(C)** Cytotoxicity [release of lactate dehydrogenase (LDH)] analyzed with technical triplicates. The addition of fresh medium is indicated in arrows.

No reports exist on bovine MuSC cultures in closed and controlled systems at higher volumes or for longer periods. We, therefore, expanded our bovine MuSC culture to a controlled benchtop stirred-tank bioreactor using commercial Cytodex^®^1 microcarriers. We performed two individual runs, both with 9,000 cells/ml, corresponding to 1,800 MuSCs per cm^2^ microcarriers in a volume of 700 ml cell cultivation medium. Approximately 30% of the culture medium was exchanged on the days indicated in figure legends (Figures 3, 4), and new Cytodex^®^1 microcarriers were added together with the medium on two timepoints. The results from the two individual runs show similar results, as described below. The two bioreactor runs were conducted with bovine MuSCs isolated from two different donors. After 31 days, we observed that glucose was almost depleted from the medium in run 1, while lactate concentration increased above the glucose concentration, even though part of the medium was replaced with fresh medium three times during the cultivation period (Figure 3A). Lactate concentration increased during the experiments and reached 0.5 g/L (4.7 mM) on day 38 (run1) (Figure 3A, left) and 0.56 g/L for run 2 (Figure 3A, right). After 1 day of initiation of the experiment (day 1), staining of the cell nucleus revealed that the bovine MuSCs were already attached to the MCs for both the runs (Figure 3B). Visual inspection showed less empty MCs over time, while the MCs with more than three cells simultaneously increased. Interestingly, the distribution of cells on the MCs was not uniform for either of the runs, and while some MCs were completely covered with cells, others were completely empty (Figure 3B, arrows and arrowheads, respectively). The addition of fresh medium seemed to promote the growth of the cell (Figure 4A) in run 1 (not measured in run 2), before the growth of the cells ceased on day 31, with a 5.7-fold increase in final ng/μl DNA. This translates to approximately 36 million cells at the end of the run. The viability remained unchanged during the experiment for run 1, indicating minimal cell death (Figure 4B). We observed a small increase in LDH released into the cell medium (Figure 4C) until day 26, before the curve flattened (except for an increase on day 26 followed by a drop on day 28).

### 3.3. Characterization of morphology and myogenic potential after expansion in lab-bench bioreactor

**Figure 5:**
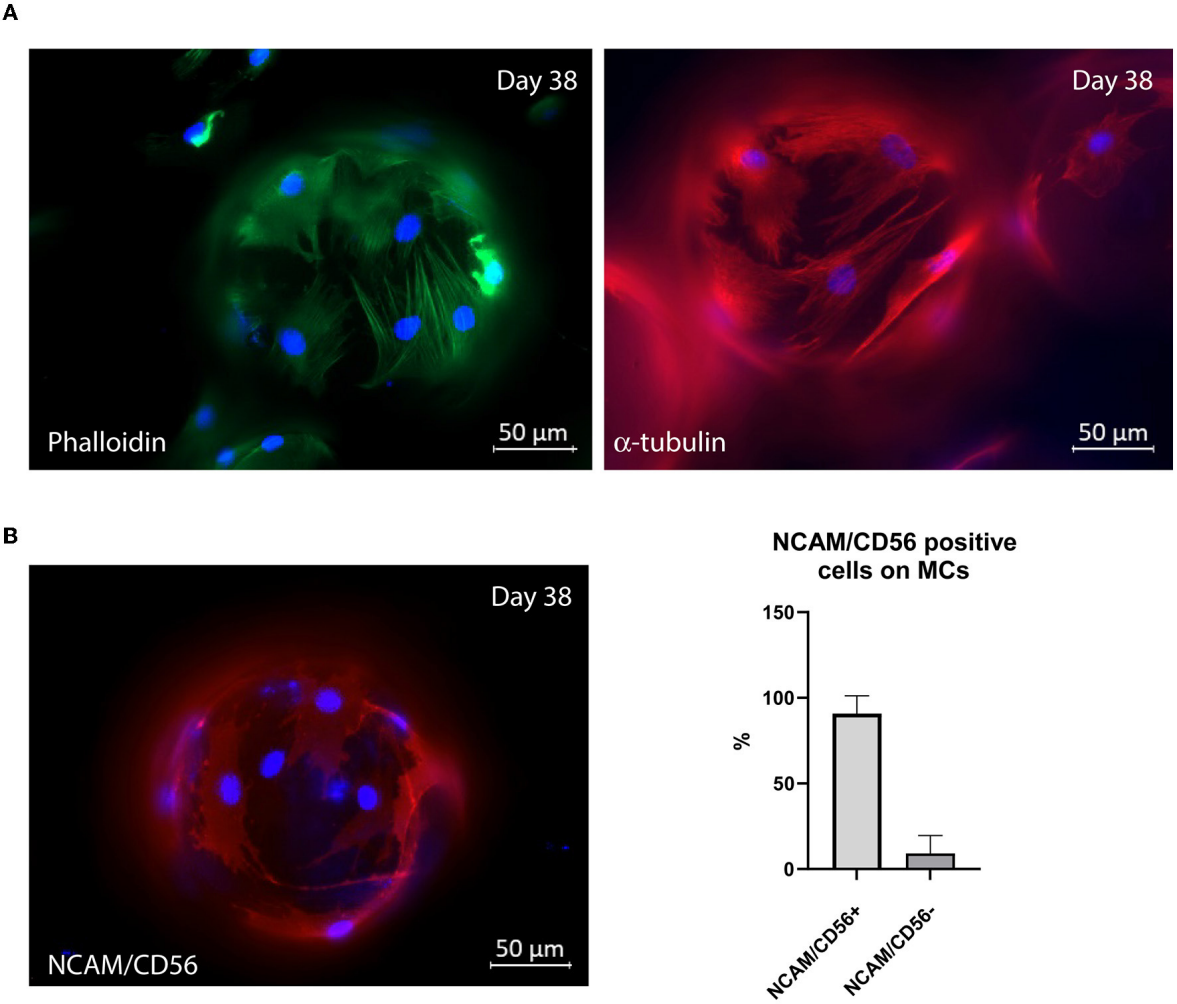
**(A)** Representative pictures showing morphology (phalloidin) and expression of α-tubulin after 38 days of cell expansion (run 1). **(B)** Representative picture showing the expression of NCAM/CD56 after 38 days of cell expansion (run 1). Six randomly captured pictures, with altogether 26 MCs, were quantified using the cell counter plugin from ImageJ to quantify the number of NCAM/DC56 positive cells on the microcarriers after 38 days of expansion. NRF bovine MuSCs on MCs were subjected to fluorescent immunostaining for assessing morphology and expression of muscle-specific markers, using Alexa-488 phalloidin and antibodies against α-tubulin and anti-NCAM/CD56. The cell nuclei were stained using NucBlue Live stain. The cells were seeded out in one replicate for each run using pooled cells isolated from five different donor animals.

MuSCs proliferating on MCs exhibited a spread and expanded cell morphology on the microcarriers (Figure 5A). Microscopic examination of MuSCs showed the expression of well-organized cytoskeletal markers such as actin and α-tubulin after 38 days of expansion (Figure 5A). Furthermore, we also observed that more than 90% of MuSCs seeded onto MCs expressed NCAM/CD56 after 38 days, confirming that they have retained their myogenic potential after prolonged expansion (Figure 5B).

**Figure 6:**
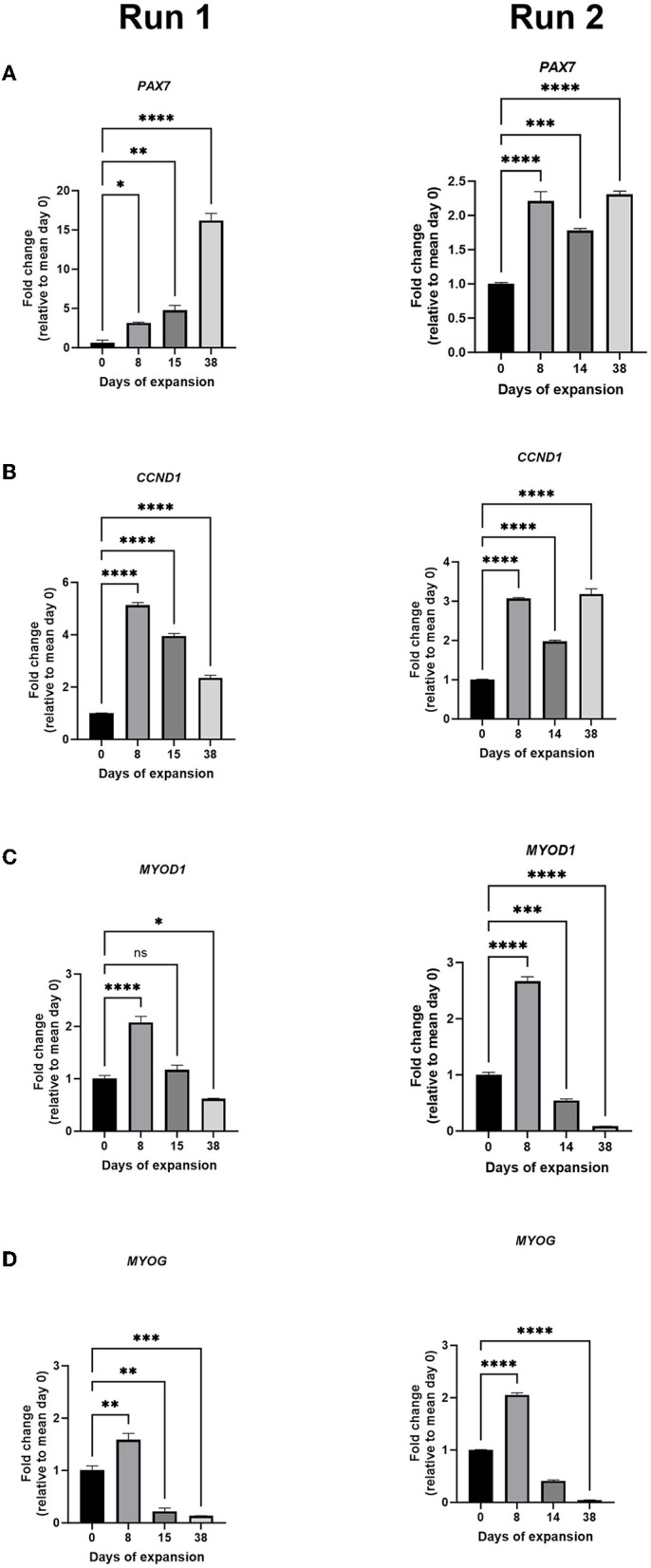
Relative gene expression (fold change) of *PAX7* **(A)**, *CCND* **(B)**, *MYOD1* **(C)**, and *MYOG* **(D)** during 38 days of MuSC expansion for run 1 (left) and run 2 (right). The data are presented as the average of technical triplicates, fold change relative to the mean of day 0. Asterisks denote significant differences between day 0 during expansion, statistics assessed using the one-way ANOVA with Dunnett's multiple comparison test. (^*^ < 0.05, ^**^ < 0.01, ^***^ < 0.001, and ^****^ < 0.0001). Long-term expansion of bovine MuSCs (38 days) in the benchtop stirred-tank bioreactor, in two biological separate replicate runs. The cells were seeded out in one replicate for each run, using cells isolated from different donor animals. For run 1, cells were isolated and pooled from five animals, while for run 2, cells were isolated from one animal.

To better characterize these long-term cultivated bovine MuSCs, we performed the RT-qPCR analysis of cells at some selected timepoints during the experiments. The relative gene expression of *PAX7* increased during the long-term expansion (Figure 6A), although the increase was much larger in run 1 compared with run 2. We also included analyses of the gene expression of *MYF5* and *DES* in the second run (Supplementary Figure S2). MYF5 is the earliest myogenic transcription factor to be expressed during myogenesis (29). Our results show that the gene expression increased on day 8, while the gene expression gradually decreased during the run (Supplementary Figure S2). The expression of critical cell cycle regulator Cyclin D1 (*CCND1*) increased during expansion on days 8 and 15 for run 1, although the expression reduced on day 38 (Figure 6B, left). For run 2, we also saw an increase on day 8 and a drop on day 14 before the expression increased again on day 38 (Figure 6B, right). The relative gene expression of myoblast determination protein 1 (*MYOD1*), a major transcription factor important for MuSC activation and involved in early differentiation, increased on day 8 for run 1, whereas the relative expression was unchanged on day 15 compared with day 0 and decreased slightly on day 38 (Figure 6C, left). For run 2, we also saw an increase on day 8 before the gene expression decreased markedly during the run (Figure 6C, right). The relative expression of *MYOG*, which is involved in later differentiation, increased on day 8 before it decreased on day 15, and the expression remained low throughout the experiment (Figure 6D, left). A similar pattern was also observed for *MYOG* in run 2 (Figure 6D, right), supported by a similar expression pattern of the differentiation marker Desmin (*DES*) (Supplementary Figure S2). We cannot rule out that some of these changes in gene expression were due to the addition of fresh medium.

**Figure 7:**
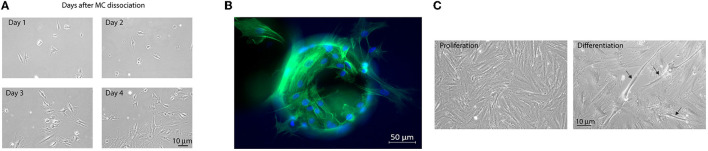
Satellite cells retained their proliferative and migratory capacity after long-term expansion and subsequent harvesting from the MCs. **(A)** Bovine MuSCs on MCs on day 34 of expansion (Run1) were dissociated from the MCs using trypsin and then seeded onto ECL-coated 6-well plates and left to proliferate for 4 days. Images were captured using a Leica LED microscope. Scale bar 10 μm. **(B)** MC/cell suspension sampled on day 29 from run 1 incubated overnight in an ECL-coated 12-well plate and stained with Phalloidin (F-actin) showed that MuSCs were able to migrate off the MCs. Images were captured using a ZEISS Axio Observer Z1 microscope. **(C)** MuSCs retained their differentiation capacity after long-term expansion and subsequent harvesting from the MCs. Bovine MuSCs on MCs on day 38 of expansion (run 2) were dissociated from the MCs using trypsin and then seeded onto ECL-coated 6-well plates and left to proliferate for 4 days (left). Proliferating MuSCs were washed with PBS and placed in a differentiation medium (DMEM, 2 % FBS, P/S, amphotericin B, and 25 pmol insulin) to induce myogenesis (right). Images were captured using a Leica LED microscope. Arrows indicate myotube formation. Scale bar 10 μm. Long-term expansion of bovine MuSCs (38 days) in the benchtop stirred-tank bioreactor, in two biological separate replicate runs. The cells were seeded out in one replicate for each run, using cells isolated from different donor animals. For run 1, cells were isolated from five animals and pooled, while for run 2, cells were isolated from one animal.

Finally, we wanted to investigate whether the cells grown on MCs in the bioreactor for 38 days could continue their proliferation on a planar system. Detached cells from the microcarriers after 34 days (run 1) and 38 days (run 2) of expansion in the benchtop stirred-tank bioreactor were clearly able to attach to the ECL-coated tissue culture plates, where they continued proliferating on a planar system (Figure 7A). In addition, it was also observed that MuSCs were able to migrate off from the MCs when a sample of MCs with cells collected on day 29 was incubated on an ECL-coated tissue culture plate for 24 h (Figure 7B) (only tested in run 1). Furthermore, we could observe that the MuSCs, which were detached after 38 days from a bioreactor to proliferate in 2D culture, were also able to form myotubes on a planar system (Figure 7C) (only investigated in run 2). Taken together, these observations show that MuSCs retain their proliferation, migration, and differentiation abilities even after long-term expansion in a stirred-tank bioreactor.

## 4. Discussion

### 4.1. Musc expansion is possible with low seeding density and low glucose in cell culture medium

Examining different cultivation parameters clearly showed that the difference between cultivation strategies could significantly increase cell expansion variability, and we observed the best increase in cell growth using high initial seeding densities and high serum concentration. Previous studies have shown that it is possible to culture bovine MuSCs on microcarriers in spinner flasks for 8–9 days (up to 250 ml) (21), and this was also confirmed by our initial experiments. Interestingly, it was possible to achieve an increase in cell growth even if the conditions were not fully optimized. Our initial spinner flask experiments showed that it was possible to achieve cell attachment and growth when using a low cell seeding density (9,000 cells/ml and 1,800 cells/cm^2^), Cytodex-1 microcarriers, and a low serum-containing medium, with the addition of the supplement Ultroser G. More importantly, we were able to expand MuSCs in the lab-bench bioreactor using the same conditions for up to 38 days. However, higher seeding densities could, on the other hand, allow us to reach maximum cell densities earlier, which could minimize production costs in terms of handling, nutrients spent, and time used. In addition, the low serum culture medium is beneficial since future cell cultivations should preferably be performed without the use of FBS (6). This is important for making cultured protein production economically feasible by removing FBS and reducing the initial cell seeding. Efficient bioprocessing requires control of critical nutrients during expansion, and glucose is a crucial nutrient as it is a significant carbon source for cell biosynthesis. High initial levels of glucose can help improve cell growth earlier during cell expansion; however, this might lead to a shift in the cell metabolism, where the MuSCs generate energy inefficiently via glycolysis rather than through mitochondrial oxidative phosphorylation (30, 31). The bovine MuSCs, in our study, were already adapted to a low-glucose medium (1g/L) to advocate for this. The growth rate in our experiment was, however, quite low compared with monolayer cultivation, where the doubling time was approximately 2.2 days (25), and one explanation could be that some of the MuSCs were in a quiescent state, rather than in a more proliferative state, suggested by the high *PAX7* expression and low *MYOD1* expression (32). In addition, in the bioreactor, the cells are more exposed to shear stress than in the 2D monolayer, which might influence their growth. The focus of our manuscript was to demonstrate the long-term maintenance of cells in a lab-bench bioreactor rather than the maximal expansion/fold increase achieved. This maintenance of MuSCs in the bioreactor could then be used to condition the cells, and once the cells have reached the appropriate levels of expression characteristics of proliferative cells, the more efficient expansion could be initiated by changing vessels, adding more MCs. Medium exchange is a way to increase MuSCs expansion, and earlier studies have suggested 50–80% medium exchange as an efficient method to save cost, to ensure constant volume during the run, to ensure sufficient supply of nutrients, and to remove waste products produced by the cells during the expansion (22, 24). In our experiment, we performed 30% medium exchange but cannot rule out that increasing this volume exchange and the number of timepoints for medium exchange could improve cell growth.

### 4.2. Ultroser G as serum replacement during MuSCs expansion

The cell culture medium composition, including Ultroser G, could also influence the growth of the cell. Ultroser G is a semi-chemically defined serum replacement containing various growth factors, including FGF, EGF, IGF, and others. Previous results demonstrated that culturing MuSCs in Ultroser G extended the life span of viable cells (33) and increased the fraction of satellite cells in primary cell cultures (34). Serum is very expensive, and the cost can be up to 95% of the total cost of the cell culture medium (35). In addition to cost, it also poses quality and reproducibility challenges and ethical and biosafety concerns due to how FBS is harvested. As such, serum supplementation is a limiting factor and cannot support sustainable large-scale cultured protein production. Extensive research in the last decade has focused on reducing and replacing FBS with a chemically defined medium (serum-free medium, SFM) as part of good cell culture practice (GCCP) (28, 36, 37). The most commercially available serum replacements show lower performance and are only suitable for a limited number of cell lines, and they may even undesirably alter the cell phenotype (2, 38). Studies conducted with importance for muscle stem cell culture have demonstrated that SFM stimulates proliferation but not to the extent of serum-containing medium (28, 39, 40). Furthermore, most of the commercially available SFMs (including Ultroser G) and serum substitute alternatives adapted to MuSCs are not food grade, and cost and performance are still an issue (36, 41). Developing a serum-free medium was out of scope for this study, but follow-up studies should examine MuSC expansion in STRs in a food-grade, chemically defined, and animal-free medium. Maintaining high purity of MuSCs without fibroblast interference is another important parameter for cultured meat production (5). High concentrations of fetal serum are not recommended for skeletal muscle cell cultures, as it has been shown to facilitate the growth of fibroblasts in culture (34). Primary cell culture isolation of bovine MuSCs usually has a purity of 90% (MuSCs vs. fibroblast contamination) (25). NCAM/CD56 is considered a reliable molecular marker for satellite cells and myoblast skeletal muscle cells in various species, including human and bovine sources (25, 42). The fact that most MuSCs in this study still expressed the muscle-specific marker NCAM/CD56 after 38 days of expansion demonstrates low contamination of fibroblasts in our system.

Adding fresh MCs mid-way of the bioprocess is another efficient method to increase cell expansion during upscaling, by increasing the surface area available to the cells. The cells can then migrate from one microcarrier to another in suspension culture, and this approach can increase cell yield and reduce process time. The success of bead-to-bead transfer highly depends on the characteristics of the surface and the cellular properties of the MCs (43). Verbruggen et al. (21) demonstrated efficient bead-to-bead transfer when new MCs were added during the experiments. It might be possible to improve and optimize bead-to-bead transfer by improving the surface properties of the MCs or by using intermitted stirring (44, 45). Furthermore, in the study by Hanga et al. (24), they showed that the addition of the microcarrier was much more efficient when combined with volume addition rather than when the volume was kept constant. In our study, we observed that after adding new microcarriers, the cells were not evenly distributed on MCs, and while some were packed with cells, others were empty by the end of the run, and this pattern remained during the expansion. The distribution of cells on MCs can be improved by intermittent stirring when fresh MCs are introduced into the process (46), but optimizing bead-to-bead transfer is beyond the scope of this study.

### 4.3. The MuSCs maintain their phenotype during the expansion in bioreactor

Efficient and long-term expansion of MuSCs must be performed under conditions that allow the cells to maintain their phenotype, i.e., migrate and proliferate. Previous experiments for expanding MuSCs *ex vivo* have been challenging because the cells started expressing myogenic transcription factors, initiating myogenesis, and differentiating, thus losing the ability to proliferate and migrate (47). Inhibiting the p38-MAPK signaling pathway has been shown as an efficient tool to maintain the ability of muscle cells to proliferate (5). During the long-term expansion of MuSCs in our experiments, the cells seemed to retain their proliferative capacity with increased gene expression of *PAX7*, even without adding p38-MAPK inhibitors. *PAX7* is a transcription factor that regulates muscle satellite cell proliferation and is a known marker of MuSCs (5, 48).

Myogenic transcription factors are important for skeletal muscle cell proliferation and the transition to the differentiation stage. The downregulation of cyclins characterizes this key step in myogenesis, and CyclinD1 is well known to regulate the activity of several transcription factors, including *MYOD1* and *MEF4* (49–51). Interestingly, we saw an increase in the mRNA expression of *CCND1* during the experiments. The interplay with cyclins, on the one hand, and *MYOD1* and its co-factors, on the other hand, play important roles during myogenesis. Previous studies have shown that the relative mRNA expression level and the protein expression level of *CCND1*/CyclinD1 decline rapidly during myogenesis (50), and no expression of *CCND1* was detected in myotubes (52). This suggests that differentiation of the bovine MuSCs was inhibited during the expansion. In addition, the ability to proliferate and migrate after enzymatic harvesting using trypsin was demonstrated in our experiments, which was supported by the ability of the bovine skeletal muscle cells to continue proliferation on a planar system, even after 38 days in the bioreactor. Furthermore, the observation that cells are able to migrate off the microcarriers when incubated overnight in an ECL-coated culture plate supports the idea of bead-to-bead transfer during cultivation.

## 5. Conclusion

Previous studies on bovine MuSCs have been performed on a smaller scale using spinner flasks for relatively short period of time, with limited control of temperature, pH, oxygenation, and nutrient consumption. Our study, on the other hand, demonstrates the long-term expansion of bovine MuSC in lab-bench bioreactors for 38 days. The primary bovine MuSCs were able to proliferate during and after long-term expansion in a benchtop stirred-tank bioreactor, supported with increased expression of the satellite cell marker *PAX7* and reduced expression of differentiation-inducing genes such as *MYOG*, without adding p38-MAPK inhibitors. Similarly, the cells retained their proliferative, migratory, and differentiation capacities after dissociation from MCs. We also showed that the results were reproducible in a separate biological run.

## Data availability statement

The original contributions presented in the study are included in the article/Supplementary material, further inquiries can be directed to the corresponding author.

## Author contributions

DT: data curation, formal analysis, methodology, writing—original draft, and writing—reviewing and editing. NS and RA: data curation, formal analysis, methodology, and writing—reviewing and editing. PM: writing—reviewing and editing. VB: methodology and writing—reviewing and editing. MP: conceptualization, funding acquisition, supervision, and writing—reviewing and editing. SR: conceptualization, data curation, formal analysis, funding acquisition, project administration, supervision, visualization, writing—original draft, and writing—reviewing and editing. All authors contributed to the article and approved the submitted version.

## References

[1] Melzener L Verzijden KE Buijs AJ Post MJ Flack JE. Cultured beef: from small biopsy to substantial quantity. J Sci Food Agric. (2020) 104:7–14. 10.1002/jsfa.10663 32662148 PMC7689697

[2] Post MJ Levenberg S Kaplan DL Genovese N Fu J Bryant CJ. Scientific, sustainability and regulatory challenges of cultured meat. Nature Food. (2020) 1:403–15. 10.1038/s43016-020-0112-z33803111

[3] Post MJ. Cultured meat from stem cells: Challenges and prospects. Meat Sci. (2012) 92:297–301. 10.1016/j.meatsci.2012.04.008 22543115

[4] Aas V Bakke SS Feng YZ Kase ET Jensen J Bajpeyi S. Are cultured human myotubes far from home? Cell Tissue Res. (2013) 354:671–82. 10.1007/s00441-013-1655-1 23749200

[5] Ding S Swennen GNM Messmer T Gagliardi M Molin DGM Li C. Maintaining bovine satellite cells stemness through p38 pathway. Sci Rep. (2018) 8:1. 10.1038/s41598-018-28746-7 30018348 PMC6050236

[6] Hubalek S Post MJ Moutsatsou P. Towards resource-efficient and cost-efficient cultured meat. Curr Opin Food Sci. (2022) 47:100885. 10.1016/j.cofs.2022.100885

[7] Judson RN Rossi FMV. Towards stem cell therapies for skeletal muscle repair. NPJ Reg Med. (2020) 5:10. 10.1038/s41536-020-0094-3 32411395 PMC7214464

[8] Pantelic MN Larkin LM. Stem cells for skeletal muscle tissue engineering. Tissue Eng Part B Rev. (2018) 24:373–91. 10.1089/ten.teb.2017.0451 29652595

[9] Bach AD Beier JP Stern-Staeter J Horch RE. Skeletal muscle tissue engineering. J Cell Mol Med. (2004) 8:413–22. 10.1111/j.1582-4934.2004.tb00466.x 15601570 PMC6740234

[10] Post MJ. Cultured beef: medical technology to produce food. J Sci Food Agric. (2014) 94:1039–41. 10.1002/jsfa.6474 24214798

[11] Tsai AC Pacak CA. Bioprocessing of human mesenchymal stem cells: from planar culture to microcarrier-based bioreactors. Bioengineering. 8, 96. 10.3390/bioengineering8070096 34356203 PMC8301102

[12] Kropp C Massai D Zweigerdt R. Progress and challenges in large-scale expansion of human pluripotent stem cells. Proc Biochem. (2017) 59:244–54. 10.1016/j.procbio.2016.09.03223515461

[13] Petry F Salzig D. Impact of bioreactor geometry on mesenchymal stem cell production in stirred-tank bioreactors. Chemie Ingenieur Technik. (2021) 93:1537–54. 10.1002/cite.202100041

[14] Allan SJ De Bank PA Ellis MJ. Bioprocess design considerations for cultured meat production with a focus on the expansion bioreactor. Front. Sust. Food Syst. (2019) 3, 44. 10.3389/fsufs.2019.00044

[15] Lawson T Kehoe DE Schnitzler AC Rapiejko PJ Der KA Philbrick K. Process development for expansion of human mesenchymal stromal cells in a 50L single-use stirred tank bioreactor. Biochem Eng J. (2017) 120:49–62. 10.1016/j.bej.2016.11.020

[16] Chen S Sato Y Tada Y Suzuki Y Takahashi R Okanojo M. Facile bead-to-bead cell-transfer method for serial subculture and large-scale expansion of human mesenchymal stem cells in bioreactors. Stem Cells Transl Med. (2021) 10:1329–42. 10.1002/sctm.20-0501 34008349 PMC8380445

[17] Moritz MSM Verbruggen SEL Post MJ. Alternatives for large-scale production of cultured beef: A review. J Integr Agric. (2015) 14:208–16. 10.1016/S2095-3119(14)60889-3

[18] Ferrari C Balandras F Guedon E Olmos E Chevalot I Marc A. Limiting cell aggregation during mesenchymal stem cell expansion on microcarriers. Biotechnol Prog. (2012) 28:780–7. 10.1002/btpr.1527 22374883

[19] Rafiq QA Ruck S Hanga MP Heathman TRJ Coopman K Nienow AW. Qualitative and quantitative demonstration of bead-to-bead transfer with bone marrow-derived human mesenchymal stem cells on microcarriers: Utilising the phenomenon to improve culture performance. Biochem Eng J. (2018) 135:11–21. 10.1016/j.bej.2017.11.005

[20] Sion C Loubière C Wlodarczyk-Biegun MK Davoudi N Müller-Renno C Guedon E. Effects of microcarriers addition and mixing on WJ-MSC culture in bioreactors. Biochem Eng J. (2020) 157:107521. 10.1016/j.bej.2020.107521

[21] Verbruggen S Luining D van Essen A Post MJ. Bovine myoblast cell production in a microcarriers-based system. Cytotechnology. (2017). 10.1007/s10616-017-0101-8 28470539 PMC5851947

[22] Hanga MP Ali J Moutsatsou P de la Raga FA Hewitt CJ Nienow A. Bioprocess development for scalable production of cultivated meat. BIT. (2020) 117:3029–39. 10.1002/bit.27469 32568406

[23] Boudreault P Tremblay JP Pépin MF Garnier A. Scale-up of a myoblast culture process. J Biotechnol. (2001) 91:63–74. 10.1016/S0168-1656(01)00291-7 11522363

[24] Hanga M. P. de la Raga FA Moutsatsou P Hewitt CJ Nienow AW Wall I. (2021). Scale-up of an intensified bioprocess for the expansion of bovine adipose-derived stem cells (bASCs) in stirred tank bioreactors. Biotechnology and bioengineering. 118, 3175–86. 10.1002/bit.27842 34076888

[25] Rønning SB Pedersen ME Andersen PV Hollung K. The combination of glycosaminoglycans and fibrous proteins improves cell proliferation and early differentiation of bovine primary skeletal muscle cells. Differentiation. (2013) 86:13–22. 10.1016/j.diff.2013.06.006 23933398

[26] Veiseth-Kent E Host V Pedersen ME. Preparation of proliferated bovine primary skeletal muscle cells for bottom-up proteomics by LC-MSMS analysis. Methods Mol Biol. (2019) 1889:255–66. 10.1007/978-1-4939-8897-6_15 30367419

[27] Messmer T Klevernic I Furquim C Ovchinnikova E Dogan A Cruz H. A serum-free media formulation for cultured meat production supports bovine satellite cell differentiation in the absence of serum starvation. Nature Food. (2022) 3:74–85. 10.1038/s43016-021-00419-1 37118488

[28] Stout AJ Mirliani AB Rittenberg ML Shub M White EC Yuen JSK. Simple and effective serum-free medium for sustained expansion of bovine satellite cells for cell cultured meat. Commun Biol. (2022) 5:466. 10.1038/s42003-022-03423-8 35654948 PMC9163123

[29] Zammit PS. Function of the myogenic regulatory factors Myf5, MyoD, Myogenin and MRF4 in skeletal muscle, satellite cells and regenerative myogenesis. Semin Cell Dev Biol. (2017) 72:19–32. 10.1016/j.semcdb.2017.11.011 29127046

[30] Mot AI Liddell JR White AR Crouch PJ. Circumventing the crabtree effect: a method to induce lactate consumption and increase oxidative phosphorylation in cell culture. Int J Biochem Cell Biol. (2016) 79:128–38. 10.1016/j.biocel.2016.08.029 27590850

[31] Whitford W. Fed-batch mammalian cell culture in bioproduction. BioProc Int. (2006) 4:1–14. Available online at: https://www.researchgate.net/publication/228627392_Fed-batch_mammalian_cell_culture_in_bioproduction

[32] Zammit PS Relaix F Nagata Y Ruiz AP Collins CA Partridge TA. Pax7 and myogenic progression in skeletal muscle satellite cells. J Cell Sci. (2006) 119:1824–32. 10.1242/jcs.02908 16608873

[33] Benders AAGM van Kuppevelt THMSM Oosterhof A Veerkamp JH. The biochemical and structural maturation of human skeletal muscle cells in culture: The effect of the serum substitute Ultroser G. Exp Cell Res. (1991) 195:284–94. 10.1016/0014-4827(91)90375-5 1649054

[34] Gaster M Beck-Nielsen H Schroder HD. Proliferation conditions for human satellite cells. The fractional content of satellite cells. APMIS. (2001) 109:726–34. 10.1034/j.1600-0463.2001.d01-139.x 11900051

[35] Specht L. An Analysis of Culture Medium Costs and Production Volumes for Cell-Beased Meat. New York, NY: The Good Food Institute (2019).

[36] van der Valk J Brunner D De Smet K Fex Svenningsen A Honegger P Knudsen LE. Optimization of chemically defined cell culture media–replacing fetal bovine serum in mammalian in vitro methods. Toxicol In Vitro. (2010) 24:1053–63. 10.1016/j.tiv.2010.03.016 20362047

[37] Kolkmann AM Van Essen A Post MJ Moutsatsou P. Development of a chemically defined medium for in vitro expansion of primary bovine satellite cells. Front Bioeng Biotechnol. (2022) 10:895289. 10.3389/fbioe.2022.895289 35992337 PMC9385969

[38] Kolkmann AM Post MJ Rutjens MAM van Essen ALM Moutsatsou P. Serum-free media for the growth of primary bovine myoblasts. Cytotechnology. (2020) 72:111–20. 10.1007/s10616-019-00361-y 31884572 PMC7002633

[39] Butler M. Serum-free media: standardizing cell culture system. Pharm Bioproc. (2013) 1:315–8. 10.4155/pbp.13.45

[40] Yamanaka K Haraguchi Y Takahashi H Kawashima I Shimizu T. Development of serum-free and grain-derived-nutrient-free medium using microalga-derived nutrients and mammalian cell-secreted growth factors for sustainable cultured meat production. Sci Rep. (2023) 13:498. 10.1038/s41598-023-27629-w 36627406 PMC9832167

[41] Rauch C Feifel E Amann EM Spötl HP Schennach H Pfaller W. Alternatives to the use of fetal bovine serum: human platelet lysates as a serum substitute in cell culture media. ALTEX. (2011) 28:305–16. 10.14573/altex.2011.4.305 22130485

[42] Boldrin L Muntoni F Morgan JE. Are human and mouse satellite cells really the same? J Histochem Cytochem. (2010) 58:941–55. 10.1369/jhc.2010.956201 20644208 PMC2958137

[43] Hassan MNFB Yazid MD Yunus MHM Chowdhury SR Lokanathan Y Idrus RBH. Large-scale expansion of human mesenchymal stem cells. Stem Cells Int. (2020) 2020:9529465. 10.1155/2020/9529465 32733574 PMC7378617

[44] Bodiou V Moutsatsou P Post MJ. Microcarriers for Upscaling Cultured Meat Production. Front Nutr. (2020) 7:10. 10.3389/fnut.2020.00010 32154261 PMC7045063

[45] Ng YC Berry JM Butler M. Optimization of physical parameters for cell attachment and growth on macroporous microcarriers. Biotechnol Bioeng. (1996) 50:627–35. 10.1002/(SICI)1097-0290(19960620)50:6&lt;627::AID-BIT3&gt;3.0.CO;2-M 18627071

[46] Wang Y Ouyang F. Bead-to-bead transfer of Vero cells in microcarrier culture. Bioprocess Eng. (1999) 21:211–3. 10.1007/s004490050665 19003145 PMC3449546

[47] Aziz A Sebastian S Dilworth FJ. The origin and fate of muscle satellite cells. Stem Cell Rev. (2012). 10.1007/s12015-012-9352-0 22278133

[48] Kuang S Kuroda K Le Grand F Rudnicki MA. Asymmetric self-renewal and commitment of satellite stem cells in muscle. Cell. (2007) 129:999–1010. 10.1016/j.cell.2007.03.044 17540178 PMC2718740

[49] Hydbring P Malumbres M Sicinski P. Non-canonical functions of cell cycle cyclins and cyclin-dependent kinases. Nat Rev Mol Cell Biol. (2016) 17:280–92. 10.1038/nrm.2016.27 27033256 PMC4841706

[50] Skapek SX Rhee J Spicer DB Lassar AB. Inhibition of myogenic differentiation in proliferating myoblasts by cyclin D1-dependent kinase. Science. (1995) 267:1022–4. 10.1126/science.7863328 7863328

[51] Wei Q Paterson BM. Regulation of MyoD function in the dividing myoblast. FEBS Lett. (2001) 490:171–8. 10.1016/S0014-5793(01)02120-2 11223032

[52] Zhang JM Wei Q Zhao X Paterson BM. Coupling of the cell cycle and myogenesis through the cyclin D1-dependent interaction of MyoD with cdk4. EMBO J. (1999) 18:926–33. 10.1093/emboj/18.4.926 10022835 PMC1171185

