# Supplementary material for: Expansion of bovine skeletal muscle stem cells from spinner flasks to benchtop stirred-tank bioreactors for up to 38 days
Source: Front Nutr. 2023 Aug 7;10:1192365. doi: 10.3389/fnut.2023.1192365 (PMC10442166; doi:10.3389/fnut.2023.1192365)
Supplement: Supplementary file 1 [file Data_Sheet_1.docx]

**Supplementary data:**

**Expansion of bovine skeletal muscle stem cells from spinner flasks to bench-top stirred-tank bioreactors for up to 38 days**

Dimitrios Tzimorotas^1^, Nina Therese Solberg^2^, R. Christel Andreassen^2^, Panagiota Moutsatsou^3^, Vincent Bodiou^3^, Mona Elisabeth Pedersen^2^, Sissel Beate Rønning^2*^

^1^ Nofima AS, Food Safety and Quality, Ås, Norway

^2^ Nofima AS, Raw Materials and Optimization, Ås, Norway

^3^ Mosa Meat BV, Watermolen 28, 6229 PM Maastricht, The Netherlands

*** Correspondence:**

Corresponding Author

Sissel Beate Rønning, Osloveien 1, 14330 ÅS, Norway, tel +47 408 56 287, sissel.ronning@nofima.no

**Supplementary Figures**


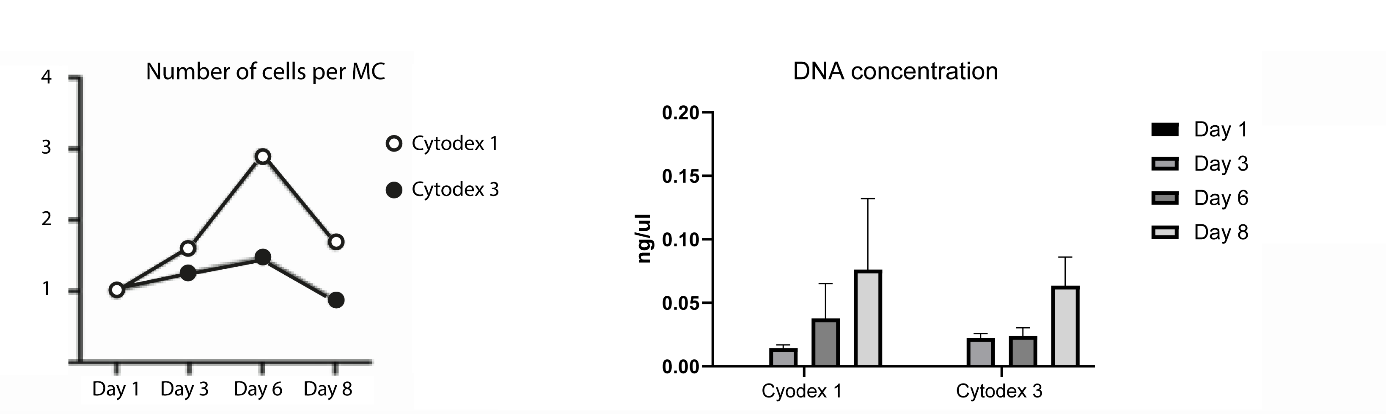


*Fig S1: Cell distribution on Cytodex©1 and Cytodex©3 MCs during expansion of bovine MuSCs for 8 days in spinner flasks. Seeding density 909 cells/cm^2^MC,20 000 cells/mL, 100 mL growth medium containing 12% FBS at 37°C. Evaluation of the number of cells per MC during cell expansion based on image analysis obtained from cell nuclei staining using Hoechst staining. Live cells were stained using NucBlue Live stain, and images were captured using a ZEISS Axio Observer Z1 microscope. A minimum of 24 pictures from each sampling day was captured to ensure unbiased quantification. To quantify the number of cells per MC, the cell counter plugin from ImageJ was used. New medium and MCS were added at day 6. The DNA concentrations were analysed using Qubit™ 1X dsDNA (HS) assay Kit with frozen samples from two replications of two independent spinner flask experiments over a time-course of 8 days, using cells isolated from one donor animal.*

*
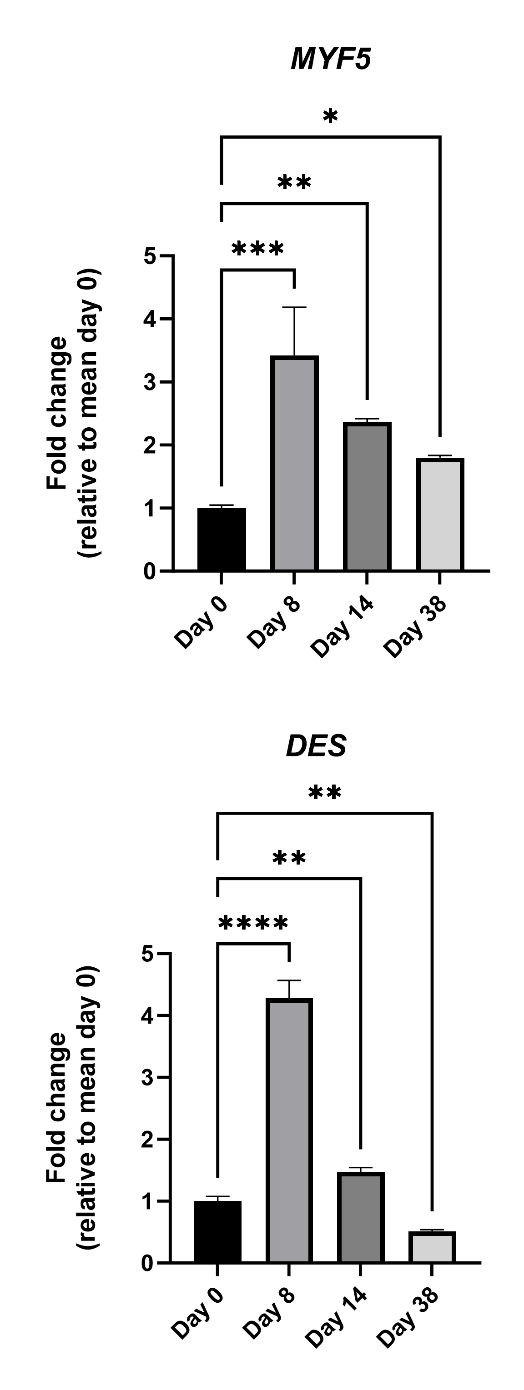
*

*Fig S2: Relative gene expression (fold change) of MYF5 and DES during 38 days of NRF MuSC expansion for Run 2. The data is presented as the average of triplicates, fold change relative to mean of day 0. Asterisks denote significant differences between day 0 and day 38 (***<0.001, ****<0.0001.), statistics assessed by one-way ANOVA with Dunnett’s multiple comparison test. The cells were seeded out in one replicate for each run, using cells isolated from one donor animals. The qPCR was analysed in technical triplicates.*
